# Investigating the prediction value of multiparametric magnetic resonance imaging at 3 T in response to neoadjuvant chemotherapy in breast cancer

**DOI:** 10.1007/s00330-016-4565-2

**Published:** 2016-09-20

**Authors:** Lenka Minarikova, Wolfgang Bogner, Katja Pinker, Ladislav Valkovič, Olgica Zaric, Zsuzsanna Bago-Horvath, Rupert Bartsch, Thomas H. Helbich, Siegfried Trattnig, Stephan Gruber

**Affiliations:** 1 High-field MR Centre, Department of Biomedical Imaging and Image-guided Therapy, Medical University of Vienna, Lazarettgasse 14, 1090 Vienna, Austria; 2 Christian Doppler Laboratory for Clinical Molecular MR Imaging, Vienna, Austria; 3 Division of Molecular and Gender Imaging, Department of Biomedical Imaging and Image-guided Therapy, Medical University of Vienna, Vienna, Austria; 4 Memorial Sloan-Kettering Cancer Center, Molecular Imaging and Therapy Service, New York, NY USA; 5 Department of Imaging Methods, Institute of Measurement Science, Slovak Academy of Sciences, Bratislava, Slovakia; 6 University of Oxford, John Radcliffe Hospital, Oxford Centre for Clinical Magnetic Resonance Research, Oxford, UK; 7 Department of Pathology, Comprehensive Cancer Center, Medical University of Vienna, Vienna, Austria; 8 Clinical Division of Oncology, Department of Medicine I, Medical University of Vienna, Vienna, Austria

**Keywords:** Breast, Neoplasms, Diffusion Weighted Imaging, Contrast-Enhanced Magnetic Resonance Imaging, Neoadjuvant Chemotherapy

## Abstract

**Objective:**

To explore the predictive value of parameters derived from diffusion-weighted imaging (DWI) and contrast-enhanced (CE)-MRI at different time-points during neoadjuvant chemotherapy (NACT) in breast cancer.

**Methods:**

Institutional review board approval and written, informed consent from 42 breast cancer patients were obtained. The patients were investigated before and at three different time-points during neoadjuvant chemotherapy (NACT) using tumour diameter and volume from CE-MRI and ADC values obtained from drawn 2D and segmented 3D regions of interest. Prediction of pathologic complete response (pCR) was evaluated using the area under the curve (AUC) of receiver operating characteristic analysis.

**Results:**

There was no significant difference between pathologic complete response and non-pCR in baseline size measures (*p* > 0.39). Diameter change was significantly different in pCR (*p* < 0.02) before the mid-therapy point. The best predictor was lesion diameter change observed before mid-therapy (AUC = 0.93). Segmented volume was not able to differentiate between pCR and non-pCR at any time-point. The ADC values from 3D-ROI were not significantly different from 2D data (*p* = 0.06). The best AUC (0.79) for pCR prediction using DWI was median ADC measured before mid-therapy of NACT.

**Conclusions:**

The results of this study should be considered in NACT monitoring planning, especially in MRI protocol designing and time point selection.

***Key Points*:**

• *Mid-therapy diameter changes are the best predictors of pCR in neoadjuvant chemotherapy.*

• *Volumetric measures are not strictly superior in therapy monitoring to lesion diameter.*

• *Size measures perform as a better predictor than ADC values.*

## Introduction

Neoadjuvant chemotherapy (NACT) is a systemic treatment that helps to reduce the size of a tumour or, in the best case, remove all tumour cells before surgery. NACT allows breast-conserving surgeries in patients with locally advanced breast cancer and often enables surgeries on initially non-operable tumours. In addition, NACT helps provide better delineation between healthy and malignant tissue during the surgery, and increases the number of patients with better postoperative recovery [1, 2]. In particular, a pathologic complete response (pCR) after NACT has been associated with a significantly better disease-free and overall survival of patients compared to a partial response [3–5]. Therefore, the non-invasive prediction of response to NACT with imaging might play an important role in potential therapy plan modifications.

Contrast-enhanced (CE)-MRI, because of its high sensitivity, is the standard method for prediction of NACT response in breast cancer. The most commonly used CE-MRI marker is the lesion size. Another marker is segmented tumour volume, which has been reported to be more predictive of response than the maximal tumour diameter [6].

Diffusion-weighted imaging (DWI) – providing a supplementary MRI contrast – is a well-established method for breast lesion characterization, with a high specificity for the detection of malignant lesions [7–10]. The apparent diffusion coefficient (ADC), derived from DWI images, reflects changes in tissue cellularity and its mean value from the lesion was found to be affected during therapy earlier than the lesion size [11, 12]. Changes of mean ADC values at an early time-point in therapy have been able to predict therapy outcome for responders, compared to non-responders, in several previous studies [11–18].

The aim of our study was to determine the optimal MRI acquisition and evaluation method, as well as the ideal time-point, using CE-MRI and DWI at 3T, to predict pCR in breast cancer patients undergoing NACT.

## Materials and methods

### Patients and therapy

Institutional review board approval and written informed consent was obtained from all patients. Forty-two breast cancer patients, 52 ± 10 years old (mean ± standard deviation, range 29 – 74), were investigated in the period from December of 2010 to December of 2013. All patients underwent baseline (BS) MRI before the NACT. During the NACT, patients were measured once or twice again. If the patient was measured twice, the first time was in the first half of the NACT and the second time in the second half.

The patient inclusion criteria were: 18 years of age or older, not pregnant, not breast feeding, histology proven cancer prior to enrolment (BI-RADS 6) with no previous treatment, and no contraindications for MR imaging or contrast agent administration.

There were two types of NACT regimens administered in 3-week long cycles:

1. taxane-based (22/42) with anthracyclines (19/22) delivered in six or eight cycles
2. anthracycline/taxane-based (20/42) consisting of four + four cycles, where anthracycline treatment and cyclophosphamide were followed by taxanes (CA – T, 16/20), or, vice versa (T – CA, 4/20).

**Fig. 1:**
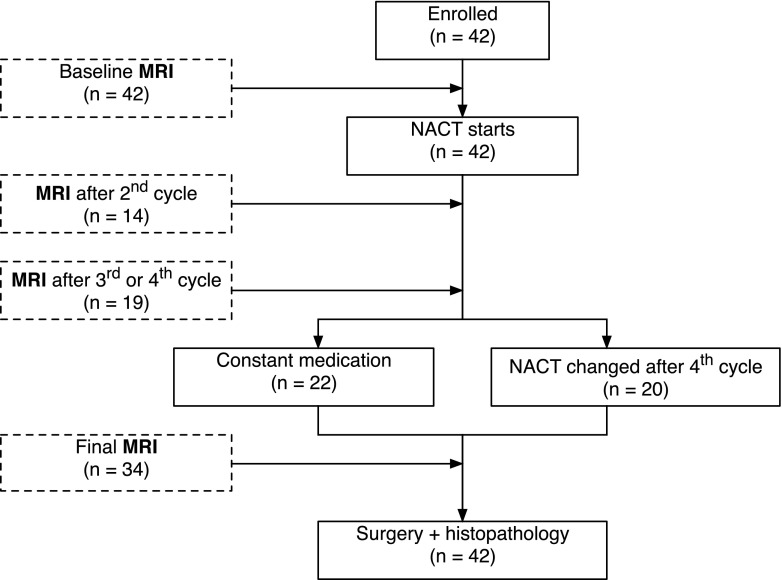
A flow chart of the study design depicting number of patient and time-points they were measured/examined. (*NACT* neoadjuvant chemotherapy)

The study flow chart with number of patients measured at different time-points and chemotherapy used is depicted on the Fig. 1.

In some patients with Her2/Neu-positive lesions (5/42), trastuzumab was used in combination with the NACT regimen. The duration of NACT therapy ranged from 84 to 168 days (three to eight cycles, median 138 days).

pCR was defined as no residual invasive or non-invasive cancer in breast tissue or in lymph nodes (ypT0 N0) on histopathology from surgical resection that was performed in all patients. The average time between the last chemotherapy and surgery was 37 ± 16 days.

### Measurements

**Fig. 2:**
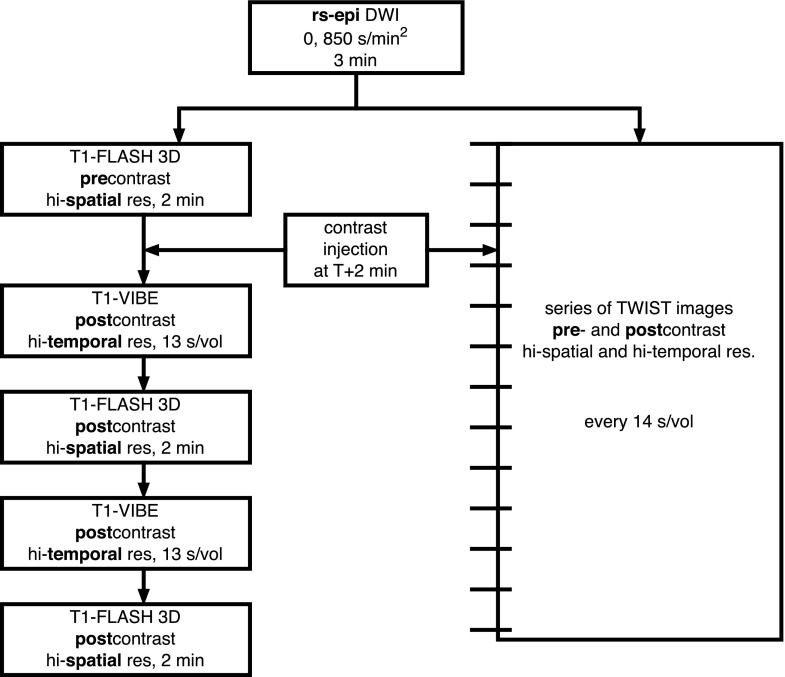
A block diagram showing the succession of sequences in the study protocol, along with sequences duration and time resolution. (*RS-EPI* readout-segmented echo planar imaging, *DWI* diffusion weighted imaging, *FLASH* fast low angle shot magnetic resonance imaging, *res*. resolution, *VIBE* volumetric interpolated breath-hold examination, *TWIST* time-resolved angiography with stochastic trajectories)

All experiments were performed on a 3 T MR system (TIM Trio, Siemens Healthcare, Erlangen, Germany) using a dedicated bilateral breast coil with four ^1^H-channels (In vivo, Orlando, FL, USA) and with patients in the prone position. First, a transversal T_2_-weighted turbo spin echo sequence with fat suppression was measured in all patients (data not used in this study). Then, DWI images were acquired with a bilateral three-scan trace using readout-segmented echo planar imaging (rs-EPI) with fat-suppression in the transversal plane and with *b*-values of 0 and 850 s/mm^2^, a repetition time (TR)/echo time (TE) of 5800/68 ms, and a total measurement time of 3 min. The rs-EPI was used, because of its advantages over single shot EPI sequence, e.g. fewer image distortions at 3 T [19]. The in-plane resolution was 1.4 × 1.4 mm^2^ with a 5-mm slice thickness. DWI was measured before contrast injection and followed by a CE-MRI sequence. Two different 3D T_1_-weighted sequences with fat suppression were used during the period of this study – both were gradient echo-based: i) a high-spatial and high-temporal resolution sequence (time-resolved angiography with stochastic trajectories – TWIST) with a temporal resolution of 14 s, spatial isotropic resolution of 1.1 mm^3^, field of view 248 × 350 mm^2^, matrix size 240 × 320, 144 slices per slab, one average, TR/TE of 6.81/2.84 ms, and a flip angle of 11° [20]; or ii) a fast low angle shot (FLASH) sequence with a duration of 2 min, isotropic 1 mm^3^ spatial resolution, field of view 320 × 134 mm^2^, matrix size 320 × 134, 96 slices per slab, one average, TR/TE of 877/3.82 ms, and a flip angle of 9°, interleaved with high temporal resolution (13 s) volumetric interpolated breath-hold examination (VIBE) imaging [21, 22]. Images from these two different protocols were considered equivalent because of similar resolution and timing of images used for tumour size evaluation. The whole protocol is also represented on the block chart in Fig. 2. Only one pre-contrast image and one maximum contrast image measured at 2 min 12 s after the contrast agent injection were used in data processing from each sequence. Gadoterate meglumine contrast agent (Dotarem, Guerbet, IN, USA) was injected intravenously as a bolus (0.1 mmol/kg of body weight) 2 min after the start of CE-MRI.

### Image analysis

The MRI was examined in consensus by two experienced radiologists, both with more than 10 years of experience in breast MRI. Tumour size was assessed based on percentage enhancement (PE) maps that were defined as PE = [(S_1_ – S_0_)/S_0_], where S_0_ and S_1_ represent the signal intensity on the pre-contrast and maximum-contrast images [2]. The volume of contrast-enhanced lesions (3Dseg) was calculated from three-dimensional segmented ROIs defined on the PE maps. The threshold used for ROI segmentation varied from 70-80 % on BS PE maps to 25-70 % on maps acquired during/after the therapy, to account for lower contrast medium uptake after the NACT, as proposed by Partridge et al. [6]. For comparison, the largest diameter of the lesion in two and three perpendicular directions was measured manually on CE images according to RECIST 1.1 [23], as the standard tumour size measure.

Non-target lesions were followed and documented as “present,” “absent,” or “unequivocal progression.” Lesions < 10 mm or pathological lymph nodes ≥ 10 mm to < 15 mm in the short axis, were considered non-measurable disease and were not considered for response assessment.

ROIs on diffusion images were defined to delineate the hyperintense areas on diffusion-weighted images (*b* = 850 s/mm^2^) [7] and corresponded to hypointense areas on the ADC map. These ROIs were marked down and then copied to the ADC maps. Two-dimensional ROIs were drawn manually on one slice with the largest tumour area as ADC data are typically processed. Necrotic areas, represented as hyperintense regions, on both DWI and ADC maps, were excluded (values higher than approx. 2 × 10^-3^ mm^2^/s). All ROIs were independently defined for each time-point.

All 3D segmented ROIs (on CE MRI and DWI) were selected using the “Grow Region” 3D segmentation with a threshold (lower/upper bounds) algorithm and processed in OsiriX® (Pixmeo, Geneva, Switzerland)/Horos^TM^ (horosproject.com). ROIs were defined only for the target lesion. The readers were blinded to pathologic results.

### Data analysis and statistics

Our statistical analysis was performed using IBM SPSS Statistics 22 (Armonk, NY, USA). In our study, the distribution of ADC values within each lesion was non-normally distributed, but since most articles report mean ADC values, we included them in our evaluation and results. Mean ADC values were calculated automatically from two- and three-dimensional ROIs. Histogram analysis was used to assess median ADC values and 15th, 25th, 50th, 75th, and 90th percentiles. The 15th and 90th percentiles were specifically reported to enable a direct comparison with literature data [24]. Only 3D ADC values, exported using the “Export ROIs” plugin in Osirix, were used for histogram analysis because of the larger number of data points.

The size measures (3Dseg, tumour diameters) and DWI measures (mean, median, and percentile of ADCs), and changes in these parameters were compared using a non-parametric Mann-Whitney U-test. In the case of paired data as, e.g., comparison between mean ADC values before and during therapy, Wilcoxon signed-rank test was used. The ability to predict pCR was assessed using the area under the curve (AUC) obtained via the receiver operating characteristics (ROC) analysis. We performed De Long’s test when comparing AUC of different measures within the same time-point and bootstrap test when comparing AUC values within different ones using the package pROC [25] in the R Project for Statistical Computing [26].

All parameter changes mentioned in the manuscript are meant to be relative to the baseline (BS) measures.

## Results

Forty-two lesions were assessed. Histological types and grades, along with receptor positivity, are listed in Table 1.

**Table 1:** Summary of clinical parameters in measured patients: histology types and grades of tumours and receptor positivity

	pCR	Non-pCR	Total number
Number of lesions	7 (17 %)	35 (83 %)	42
Age, years^a^	55 ± 11	52 ± 10	52 ± 10
Tumour histology			
IDC	7	34	41
ILC	0	1	1
Histological grade			
1	0	1	1
2	0	11	11
3	7	23	30
Oestrogen receptor			
Positive	3 (43 %)	24 (69 %)	27 (64 %)
Negative	4 (57 %)	11 (31 %)	15 (36 %)
Progesterone receptor			
Positive	0 (0 %)	13 (37 %)	13 (31 %)
Negative	7 (100 %)	22 (63 %)	29 (69 %)
HER-2/neu			
Positive	1 (14 %)	4 (11 %)	5 (12 %)
Negative	6 (86 %)	31 (89 %)	37 (88 %)
Oestrogen & Progesterone			
Positive	1 (14 %)	13 (37 %)	14 (33 %)
Triple-negative	3 (43 %)	9 (26 %)	12 (29 %)
Tumour diameter			Mean
Pre-treatment^a^ (BS)	4.7 ± 2.3	5.7 ± 3.0	5.6 ± 3.0

Note: *pCR* pathologic complete response, *IDC* invasive ductal carcinoma, *ILC* invasive lobular carcinoma. Percentages are from the number of patients in the specific pCR/non-pCR/total group

^a^Mean ± standard deviation

AUC values from the ROC analysis for pCR prediction (using i.e., largest tumour diameter, 2D and 3D diameters, 3Dseg) at the initial time-point (BS) and during the chemotherapy are displayed in Table 2.

**Table 2:** Area under the curve from the ROC analysis of pathologic complete response prediction using different MRI measures

AUC	Baseline	After 2 cycles	After 3-4 cycles	After 5 cycles
Size measure: number of lesions in the analysis (pCR)	41 (7)	13 (3)	18 (3)	36 (6)
Lesion size				
Maximal diameter	0.563			0.821
2D diameter	0.580	0.667	0.889	0.761
3D diameter	0.626	0.633	0.933	0.761
3Dseg	0.527	0.600	0.889	0.788
Size ratio (TP/BS)				
2D diameter		0.833	0.889	0.817
3D diameter		0.833	0.933	0.811
3Dseg		0.700	0.867	0.727
ADC measure: number of lesions in the analysis (pCR)	42 (7)	14 (3)	19 (3)	34 (6)
2D ADC values				
Mean	0.637	0.667^a^	0.542	0.800
3D ADC values				
Mean	0.633	0.697^a^	0.500	0.743
Median	0.635	0.788^a^	0.563	
15th percentile	0.669	0.697^a^	0.583	
25th percentile	0.673	0.636^a^	0.521	
75th percentile	0.527	0.697^a^	0.500	
90th percentile	0.531	0.636^a^	0.521	
Δ 3D ADC (TP-BS)				
Mean		0.606	0.729	0.577
Median		0.788	0.750	
15th percentile		0.667	0.771	
25th percentile		0.788	0.750	
75th percentile		0.515	0.646	
90th percentile		0.576	0.729	

Note: *pCR* pathologic complete response, Δ difference of, *3Dseg* 3D segmented tumour size, *TP* time-point, *BS* baseline; the numbers in parenthesis correspond to the number of pCR patients in that group

^a^in ROC analysis, using real ADC values, smaller values were considered positive for pCR prediction in BS, after three to four cycles and after five to eight cycles; however, higher values were considered positive for pCR prediction in data measured after two cycles

Changes in 2D diameter, ADC values, initial diameter, response, and NACT regimens for all lesions are listed in Table 3.

**Table 3:** Changes in 2D diameter and ADC values, initial diameter, response, and neoadjuvant chemotherapy regimens in all lesions. The top half of the table includes lesions with pathological complete response (pCR), and below are lesions without (non-pCR)

				2D diameter size change from BS [%]		Mean ADC values [×10^-3^ mm^2^/s]	
Patient number	NACT regimen	Response	Initial diameter at BS (cm)	2nd-4th cycle	after 5th cycle	2nd-4th cycle	after 5th cycle
1	TA	pCR	2.34	n/a	-100	n/a	n/a
2	CA - T	pCR	8.58	-40	n/a	1.59	1.49
3	CA - T	pCR	3.09	-43	-64	1.37	0.91
4	CA – T	pCR	3.04	-28	-78	1.39	1.18
5	TA, Tr	pCR	4.42	-100	-100	1.24	n/a
6	TA	pCR	6.9	-100	-100	1.09	0.97
7	T - CA	pCR	4.83	-73	-100	0.92	0.95
8	CA - T	non-pCR	2.98	-91	-100	1.25	n/a
9	CA - T, Tr	non-pCR	6.85	n/a	-32	n/a	0.87
10	TA	non-pCR	9.15	-7	-38	1.54	1.53
11	TA	non-pCR	2.63	-22	-100	1.44	2.08
12	T - CA	non-pCR	3.12	-28	-55	1.13	1.53
13	CA - T	non-pCR	10.28	-28	-28	1.89	1.67
14	TA	non-pCR	4.5	-19	-100	0.99	1.61
15	CA - T	non-pCR	6.35	n/a	-34	n/a	1.04
16	TA	non-pCR	4.1	-17	-35	1.03	1.20
17	TA, Tr	non-pCR	10.64	-48	-64	1.22	1.51
18	CA - T	non-pCR	4.39	n/a	-47	n/a	1.58
19	CA - T, Tr	non-pCR	6.84	n/a	-100	n/a	n/a
20	T - CA	non-pCR	5.29	-86	-71	1.76	1.47
21	CA - T	non-pCR	10.11	-3	-100	1.13	2.03
22	TA	non-pCR	2.98	-38	-57	0.98	1.23
23	TA	non-pCR	9.88	n/a	-100	n/a	1.38
24	TA	non-pCR	2.97	n/a	-51	0.76	1.17
25	CA - T	non-pCR	6.51	-100	n/a	1.07	n/a
26	TA	non-pCR	2.97	-32	n/a	1.31	n/a
27	TA	non-pCR	n/a	n/a	n/a	0.89	0.87
28	TA	non-pCR	4.02	-34	-100	0.96	1.33
29	TA	non-pCR	4.82	n/a	-29	n/a	1.15
30	T - CA	non-pCR	2.05	-23	-21	1.60	0.99
31	TA	non-pCR	2.36	-46	-100	0.97	n/a
32	TA	non-pCR	7.05	-12	-7	1.47	1.86
33	CA - T	non-pCR	5.38	-18	n/a	1.27	n/a
34	TA	non-pCR	4.49	n/a	-20	n/a	0.91
35	TA	non-pCR	7.58	1	-9	1.12	1.43
36	CA - T	non-pCR	2.39	17	4	0.98	1.00
37	CA - T	non-pCR	8.88	-23	-33	1.10	1.54
38	T, Tr	non-pCR	2.4	-25	-28	1.00	1.01
39	TA	non-pCR	10.32	-1	n/a	1.46	n/a
40	TA, Tr	non-pCR	3.52	-20	-28	1.51	1.27
41	CA - T	non-pCR	12.48	0	-4	0.80	0.98
42	CA - T	non-pCR	6.55	33	64	1.38	1.41

Note: *T* taxane, *A* anthracycline, *C* cyclophosphamide, *Tr* traustuzumab, *NACT* neoadjuvant chemotherapy, *n/a* value missing

### Comparison of 2D and 3D ADC

Three-dimensional ADC values from all time-points and patients were not significantly different from 2D ADC (*p* = 0.061). In general, mean ADC values from 3D ROIs performed better or similarly to 2D ROIs in pCR prediction with higher/similar AUCs in ROC analysis (see Table 2). Moreover, 3D ADC ROIs have the advantage of more single ADC values for histogram analysis. Therefore, all comparisons of ADC values are provided from 3D ROIs, if not stated otherwise.

**Fig. 3:**
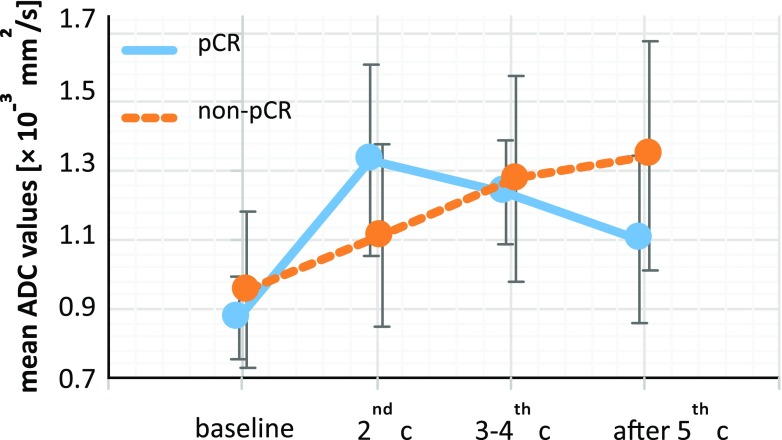
Plot depicting mean ADC values of pathological complete responders (pCR) and non-pCR before (baseline) and during (after the 2nd, 3rd and 4th, and 5th cycle) the neoadjuvant chemotherapy

There was no significant difference between pCR and non-pCR lesions using mean ADC values at any time-point (BS: *see above*, after second cycle: *p* = 0.368, after third and fourth cycle: *p* = 0.999, after fifth cycle: *p* = 0.093). However, it is still important to note the behaviour of the mean ADC values development during the NACT, which is depicted on Fig. 3. Moreover, the ADC values from 2D ROIs measured after the fifth cycle were significantly lower in the pCR group (*p* = 0.034). We were motivated to investigate this difference because of the high AUC value (0.800) at this time-point (see Table 2). In comparison, the difference between the pCR and non-pCR groups using 2D ROIs was not significant for other time-points (*p* = 0.272 at the BS, 0.456 after the second cycle and 0.875 after the third and fourth cycle). However, there was no significant difference in AUCs between any time-point or when comparing different methods.

**Fig. 4:**
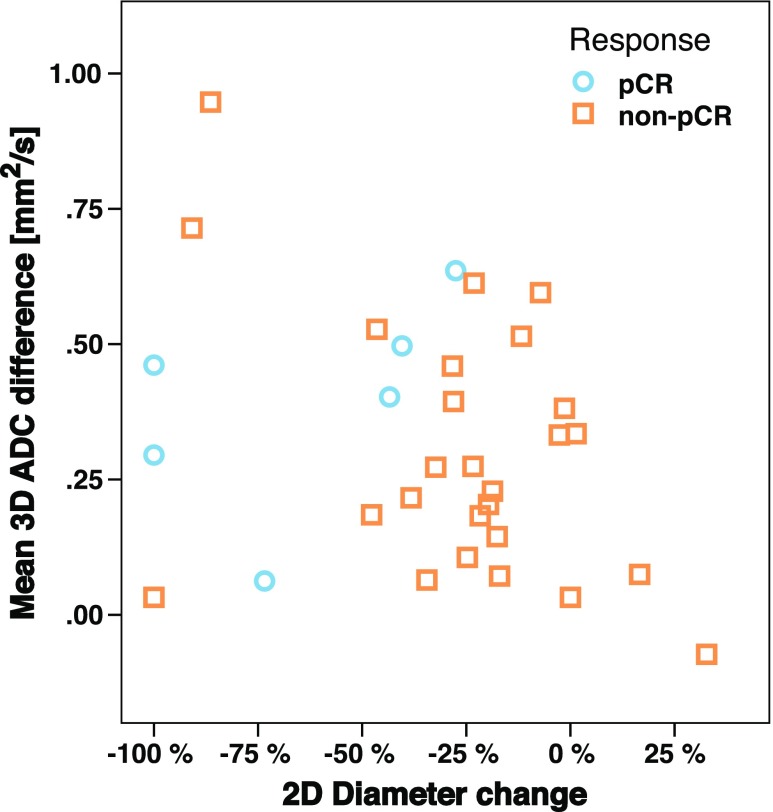
A scatter plot depicting the relation of median 3D ADC change and 2D diameter change in the first half of the therapy (after 2nd or 3rd or 4th cycle) from the baseline measurement. The size change shows a good predictor for pCR because of the majority of non-pCR cases are distributed on the left side of the plot. However, the ADC values for pCR cases are distributed equally along with non-pCR on the y-axis; therefore, they show little to no contribution to the pCR prediction at this time-point

The potential added benefit of ADC values for pCR prediction during second, third, and fourth cycle was investigated on a scatter plot by correlating 2D diameter change with median 3D ADC values (Fig. 4).

**Fig. 5:**
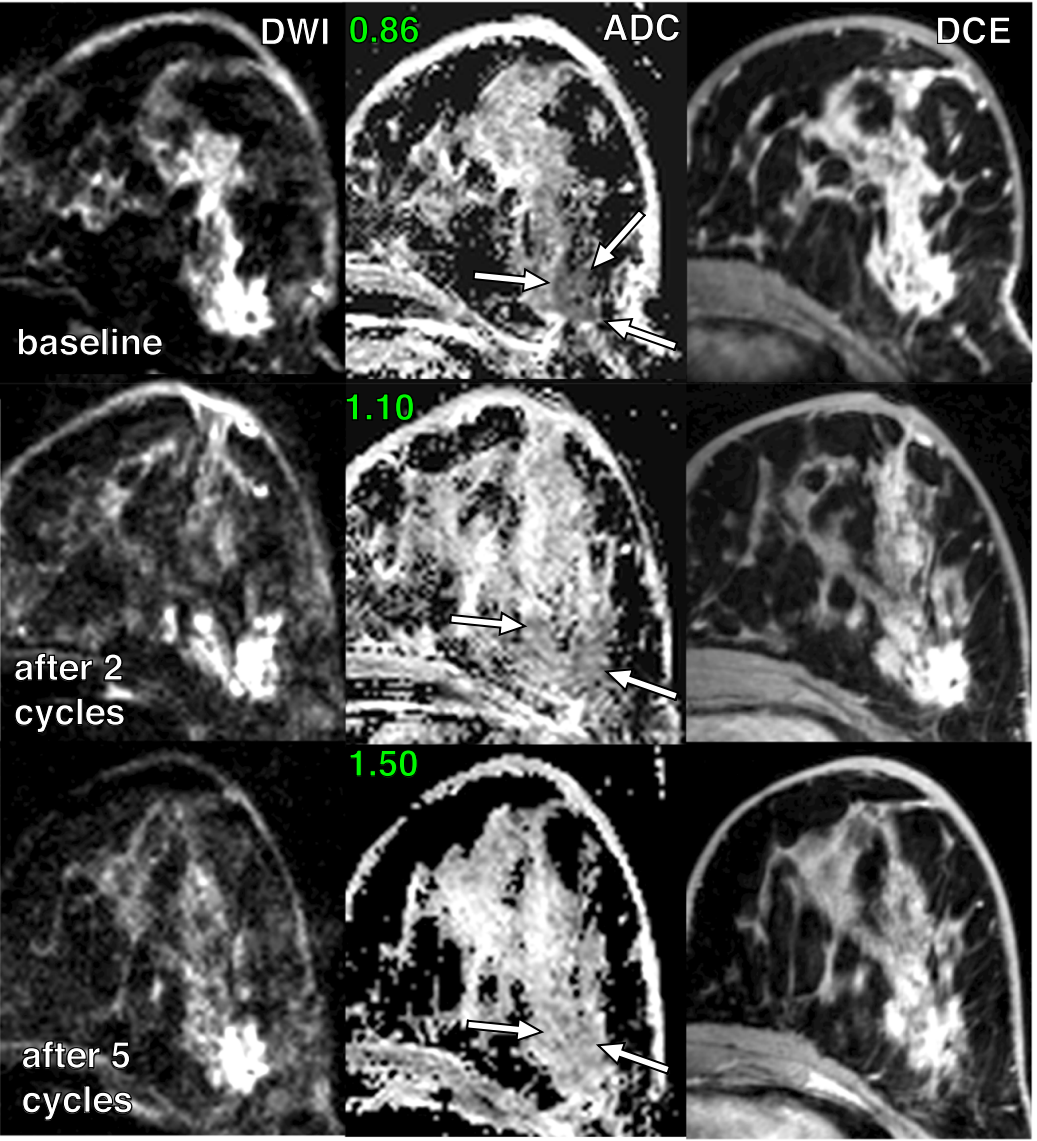
Examples of DWI and CE MRI in a 39-year-old patient with invasive ductal carcinoma and stable disease after neoadjuvant chemotherapy (non-responder). Mean ADC values (×10^-3^ mm^2^/s) measured before and during the therapy are depicted next to the corresponding ADC map, with a region of interest surrounding the lesion (*white arrows*)

**Fig. 6:**
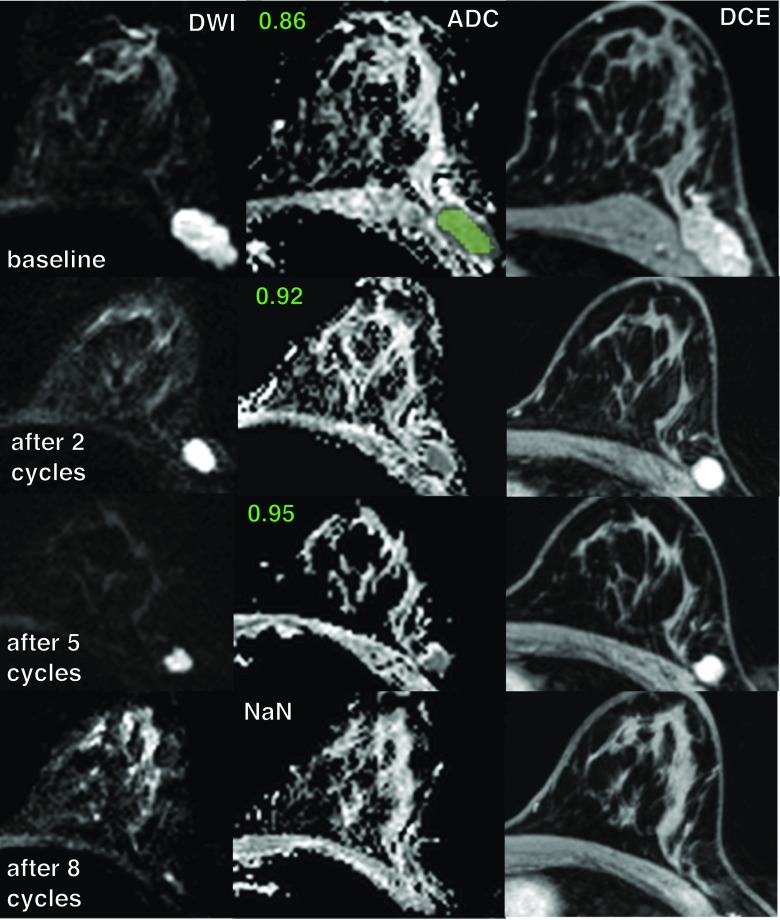
Examples of DWI and CE MRI in a 37-year-old patient with invasive ductal carcinoma and pathologic complete response to neoadjuvant chemotherapy. Mean ADC values (×10^-3^ mm^2^/s) measured before and during the therapy are depicted next to the corresponding ADC maps. An example of a segmented region of interest fills the lesion (green colour) in the baseline ADC map. *NaN* value missing

Selected examples of a false-positive case with higher ADC values during and after NACT in a non-responder and a false-negative case, with subsequently low ADC values during and after NACT in a pCR lesion, are shown in Figs. 5 and 6, respectively.

### Baseline measurements

All patients were included in baseline data analysis. The tumour diameter ranged from 2.1 to 12.5 cm before therapy measured on CE-MRI.

There was no significant difference between the pCR and non-pCR groups in all BS tumour size measures (*p* = 0.843 for 3Dseg, 0.388 for 3D diameter, and 0.530 for 2D diameter).

The mean ADC values were not significantly different for pCR in comparison with non-pCR (*p* = 0.287) and the values were: 0.87 ± 0.12 × 10^-3^ mm^2^/s in pCR and 0.96 ± 0.23 × 10^-3^ mm^2^/s in non-pCR.

### After the second cycle

The size diameter change was more prominent on average within pCR cases than in non-pCR tumours, but this difference was not significant (*p* = 0.371 using 3Dseg, 0.112 using 3D diameter, and 0.112 using 2D diameter of tumour).

The mean ADC values measured after the second cycle were significantly higher than at BS (*p* = 0.008). The mean ADC values after second cycle were as follows: pCR – 1.33 ± 0.28; and non-pCR – 1.13 ± 0.26 × 10^-3^ mm^2^/s.

### After the third and fourth cycles

The tumour diameter change from the baseline started to be significantly higher for pCR cases when compared with non-pCR (*p* = 0.017 in 3D diameter and 0.039 in 2D diameter). However, there was no significant difference between pCR and non-pCR using 3Dseg (*p* = 0.056).

The mean ADC values were again significantly higher from BS (*p* < 0.001). Mean ADC values after third and fourth cycle were: 1.24 ± 0.15 in pCR and 1.28 ± 0.30 × 10^-3^ mm^2^/s.

### After the fifth cycle

There was again a significant difference between pCR and non-pCR using 2D and 3D tumour diameter change (*p* = 0.016 for 3D diameter and 0.014 for 2D diameter) and the difference was not significant for 3Dseg (*p* = 0.116).

The mean ADC values after the fifth cycle were significantly higher than the BS (*p* < 0.001). The mean ADC values after fifth cycle were as follows: pCR – 1.10 ± 0.24; non-pCR – 1.34 ± 0.33 × 10^-3^ mm^2^/s.

## Discussion

In this study, we measured breast cancer patients at several time points before and during NACT using CE-MRI and DWI at 3 T. We assessed the ability to predict pCR using three tumour size measures based on CE-MRI in addition with two different ROI definition approaches on ADC maps. Our results show the advantage of tumour size measures for therapy monitoring, mostly during the first half of NACT. Compared to size measures, we found that ADC values were not good enough for NACT prediction, not even after the second cycle of NACT.

In a comparable study at 1.5 T by Fangberget et al. [14], DWI and CE-MRI was performed in 31 patients at three time points: baseline, after the fourth cycle, and before the surgery. This study found ADC values, tumour size, and tumour size reduction after four cycles of chemotherapy to be strong predictive markers for pCR. They found AUC for pCR prediction using ADC values to be 0.80 and the ADC values were significantly higher in pCR in the middle of the therapy, which was found in our case at an earlier time-point and the difference was not significant. In the study, even patients with remaining DCIS were included in the pCR group, the ADC ROIs were drawn on ADC maps, and they used single-shot EPI with 1.5 times smaller resolution (1.5 × 1.5 × 4 mm^2^), which could have caused increased partial volume effect from healthy/necrotic tissue. These factors all together could be the source of differences of their results from ours.

We found that tumour diameter measurement at the mid-therapy time-point (after the third or fourth cycle) is more predictive for pCR than at other time-points. The highest AUC values (>0.9) were found for 3D diameter changes measured after the third and fourth cycle of NACT. This is in agreement with previous reports by Hylton et al. [2]. In this study, they used data from 216 patients and included only tumours bigger than 3 cm (we had five cases with a diameter of less than 2.5 cm), and DCIS was part of their pCR group. In our study, DCIS lesions were considered as non-pCR because pCR, defined as no residual invasive or non-invasive cancer, was found to be associated with, highly favourable outcome, compared to other groups in a study by von Minckwitz et al. [3]. Furthermore, Hylton et al. found the segmented volume to perform better in pCR prediction at the early time-point (after the first cycle) than tumour diameter, but in our data, diameter measures were always more advantageous or similar to tumour volume.

Additionally, we have found that if the baseline data would not be present, the tumour size alone – measured after the second cycle – was much less predictive for pCR than the tumour size measured later in the therapy. In contrast, the change in tumour size from the baseline performed well in predicting the pCR outcome at every time point.

In a study by Partridge et al. [6], the baseline tumour volume, diameter, and tumour volume change after NACT were associated with the length of recurrence-free survival, but early (after the first cycle) tumour volume and largest diameter were not. pCR was found to be associated with a highly favourable outcome [3], which would suggest an association with recurrence-free survival, too. However, in our study, all baseline tumour size measures were not sufficient to efficiently predict pCR (AUC values of 0.527 – 0.626), and there was no difference between pCR and non-pCR patients using baseline size measures.

ADC values measured before NACT were lower in the pCR group, although not significantly, and were hardly able to predict pCR (highest AUC of 0.669 for the 15th percentile). In contrast, two other studies found significantly higher pre-treatment ADC values in responders when compared to non-responders [16, 27]. Wilmes et al. [24] found that lower pre-treatment ADC metrics were generally found in responders to therapy, but the difference was significant only for high-resolution DWI. This could be supported by the hypothesis that higher ADC values are linked to tissue necrosis, characterized by hypoxia, acidity, and poor perfusion, which might account for the resistance to treatment [28, 29]. Several other studies found no correlation with tumour size change or pathologic complete response [11, 30, 31], which is in accordance with our results. However, a study by Richard et al., found ADC values to be a good predictor of non-pCR considering breast cancer subtypes [32].

We found that ADC values in tumours increased during chemotherapy, which is in agreement with the literature [12].

ADC metrics and their changes during the first half of the therapy were of limited value in predicting pCR (AUC values ranged from 0.5 to 0.788), when compared with size measures at the same time-points. Interestingly, the DWI measurement after the second cycle was the only time at which mean ADC values were higher in the pCR group than in the non-pCR group. While in the non-pCR group, the mean ADC values tended to increase or stay similar to the time-points before, in pCR group there is an increase in ADCs after the second cycle and then the values decrease again. It is possible that better results would have been obtained if ADCs were assessed at an earlier time-point. ADC assessment at the second cycle could have been too late to detect necrotic changes caused by the chemotherapy and represented by higher diffusivity. Lesions could have already started to transform into fibrotic tissue. Possible evidence of outgoing fibrosis can be deducted from high AUC obtained using mean ADC values from 2D ROIs after the fifth cycle, where the ADC values in pCR lesions were significantly lower than in non-pCR that could be caused by fibrosis in the responding tumours. Moreover, there was not a big difference between 2D and 3D ADC values, when used for pCR prediction. This could have been caused by the non-isotropic resolution of the DWI images (5 mm slice thickness).

Our semi-automated segmentation method provided mean ADC values comparable to manually delineated 2D ROIs. This ROI determination technique is faster than manual delineation. Moreover, 3D segmented ADC ROIs were more convenient for histogram analysis, because there is higher number of ADC values per each lesion. Furthermore, median ADC values from 3D data have proved to be better in pCR prediction than mean values.

Other factors that could contribute to different results, when compared to the literature, include: different DWI acquisition parameters, ROI delineation, and differences in tumour size assessment. More data can help to understand better the benefits of integrating mean (median) ADC values as an additional imaging biomarker for NACT monitoring. Moreover, the limitation of this study was that the smaller number of patients at earlier time-points after the start of NACT and that patients were not all measured at all time-points. This was not possible because of the management complications caused by NACT side effects. Moreover, patients measured after the fifth cycle were not all measured after the end of the therapy. Another factor influencing tumour size estimation could be delay in enhancement of the tumour, making parts of the tumour not yet visible at the time of the post-contrast measurement.

In conclusion, the results of this study suggest that size changes assessed at an earlier time-point predict pCR in NACT better than later measurements. No advantage for therapy monitoring was found in using segmentation based volumetric measures when compared with the standard 2D diameter. If measured in the middle of the therapy, DWI measurement is less capable in therapy outcome prediction than size change measures.

## Abbreviations

3Dseg: Three-dimensional segmented tumour size
ADC: Apparent diffusion coefficient
BS: Baseline
CE MRI: Contrast-enhanced MRI
DCIS: Ductal carcinoma in situ
DWI: Diffusion-weighted imaging
IDC: Invasive ductal carcinoma
ILC: Invasive lobular carcinoma
NACT: Neoadjuvant chemotherapy
pCR: Pathologic complete response
ROI: Region of interest
